# Delayed anterolateral radial head dislocation secondary to radial shaft fracture malunion: A case report

**DOI:** 10.1097/MD.0000000000028661

**Published:** 2022-02-11

**Authors:** Sung Il Wang, Seung Cheol Lee

**Affiliations:** Department of Orthopaedics Surgery, Jeonbuk National University Medical School, Research Institute for Endocrine Sciences and Research Institute of Clinical Medicine of Jeonbuk National University–Biomedical Research Institute of Jeonbuk National University Hospital, 567 Baekje-ro, Dukjin-gu, Jeonju, Republic of Korea.

**Keywords:** dislocation, malunion, radial head, radius shaft

## Abstract

**Rationale:**

Traumatic radial head dislocation (RHD) can occur due to hyperpronation injury with sequential disruption of the annular ligament, quadrate ligament, and the interosseous membrane. Although studies have shown that traumatic RHD is generally associated with Monteggia fracture-dislocation, traumatic RHD occurring with ipsilateral radial shaft fractures has rarely been reported. Delayed RHD secondary to the malunion of isolated radial shaft fractures is extremely rare.

**Patient concerns:**

We report the case of a 12-year-old right-handed boy with progressive pain and limited range of motion in the right elbow.

**Diagnosis:**

The patient was diagnosed with delayed RHD associated with radial shaft fracture malunion.

**Interventions and outcomes:**

A corrective osteotomy was performed at the site of malunion with open reduction of the radial head using an extensile lateral approach. The annular ligament was disrupted. Forearm rotation causes radial head subluxation Therefore, the Bell Tawse procedure was additionally performed to reconstruct the annular ligament by turning down a strip of triceps tendon and anchoring it around the radial neck.

**Lessons:**

Malunion of the radial shaft can cause delayed RHD with a limited elbow range of motion. Annular reconstruction using a strip of the triceps tendon and corrective osteotomy of the radial shaft with an extensile lateral approach may be useful for treating this rare entity or situation.

## 1 Introduction

Traumatic radial head dislocation (RHD) can occur due to hyperpronation injury with sequential disruption of the annular ligament, quadrate ligament, and the interosseous membrane. Although studies have shown that traumatic RHD is generally associated with Monteggia fracture-dislocation,^[1]^ traumatic RHD occurring with ipsilateral radial shaft fractures has rarely been reported.^[2,3]^ Delayed RHD secondary to the malunion of isolated radial shaft fractures is extremely rare. In the English-language literature, only 2 such cases have been reported.^[4,5]^ Therefore, there has been no report on a useful treatment technique in situations where annular ligament reconstruction is required because the radial head is in an unstable state even after corrective osteotomy of the malunited radius. Herein, we report a case of delayed RHD associated with malunion of a radial shaft fracture in a 12-year-old-boy. The results of the present study suggest that annular reconstruction using a strip of the triceps tendon and corrective osteotomy of the radius with an extensile lateral approach may be useful for treating this rare entity or situation.

## 2 Consent

The patient signed an informed consent form for publishing this case report and accompanying images. Ethical approval for this study was waived by the ethics committee of Jeonbuk National University Hospital because this study was a case report, and the number of patients was less than 3.

## 3 Case report

**Figure 1:**
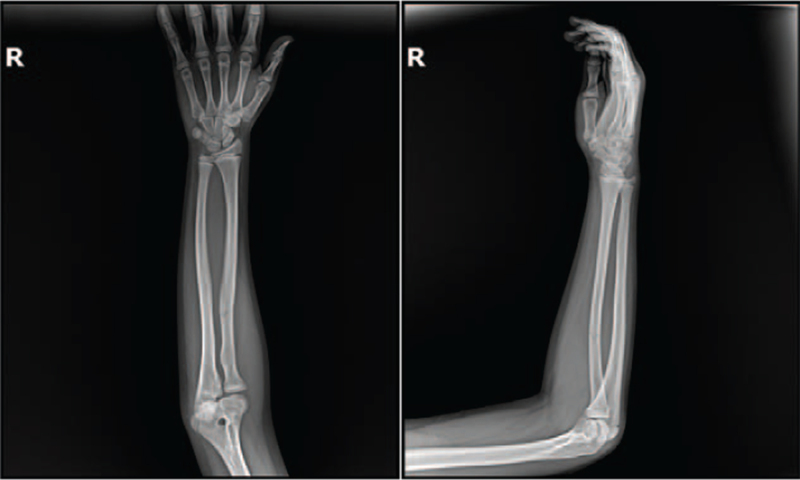
Clinical photographs taken preoperatively and at the last follow-up. A) In preoperative photographs, active range of motion is 80° of flexion and 5° of extension for the right elbow. The range for supination of the right forearm is limited at 5°. B) In last follow-up photographs, active range of motion is 105° of flexion, 0° of extension, and 85° of supination for the right elbow.

A 12-year-old right-handed boy presented to our clinic complaining of progressive pain and limited range of motion (ROM) in the right elbow. The patient complained of difficulty in performing tasks such as washing the face, eating something, or cleaning the perineal area after bowel movement. He was diagnosed with a fracture of the right radial shaft after falling from a height of 2 meters 16 months previously. The fracture was treated by cast immobilization with the elbow at 90° flexion in a local clinic. Physical examination at our clinic revealed a carrying angle of 26° valgus at the elbow on the right and 16° on the left. Active and passive ranges of motion were 80° of flexion and 5° of extension for the right elbow (Fig. 1A). The range for pronation was 65° on the right, and, supination was 5°.

**Figure 2:**
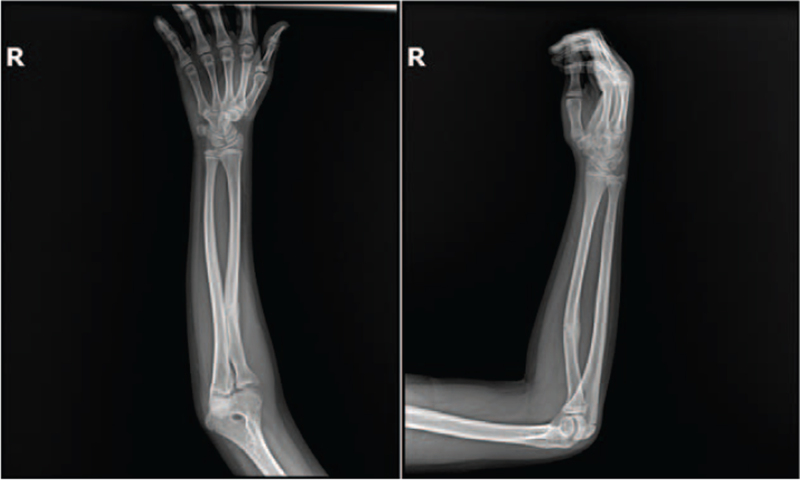
Radiographs of the right forearm performed at the time of injury. Anteroposterior and lateral radiographs showing linear fracture without displacement of the radius at the proximal third, but a radial fracture with an anterior convex deformity of 8°. No radial head dislocation was observed.

Radiography of the right forearm performed at the time of injury showed a linear fracture without displacement of the radius at the proximal third, although a radial fracture with an anterior convex deformity of 8° was found. No dislocation of the radial head was observed (Fig. 2).

**Figure 3:**
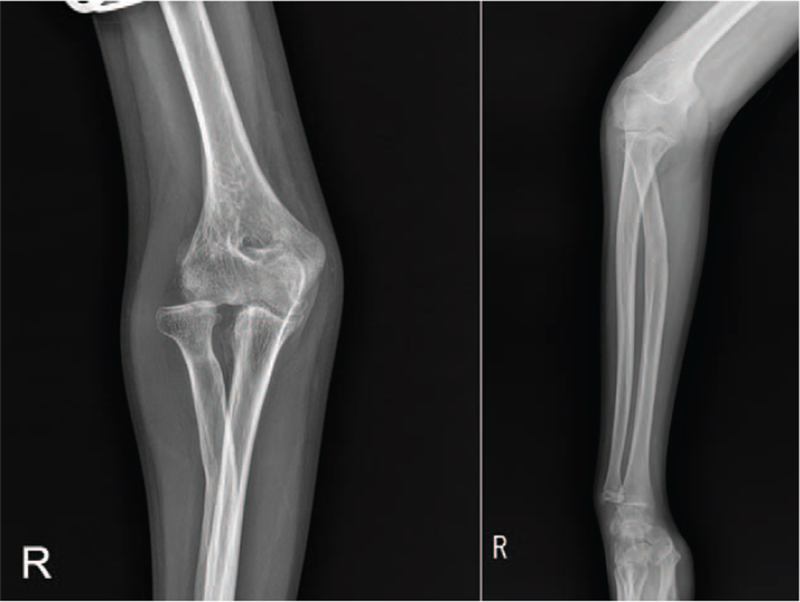
Radiographs performed at 6 wks after injury. The anterior convex deformity of the radial shaft increased to 16°, although radial head dislocation was not apparent radiographically.

Six weeks after the injury, the anterior convex deformity of the radial shaft increased to 16°, although dislocation of the radial head was not apparent radiographically (Fig. 3). The cast was removed, and active elbow and forearm motion was permitted. The patient did not visit a local clinic.

**Figure 4:**
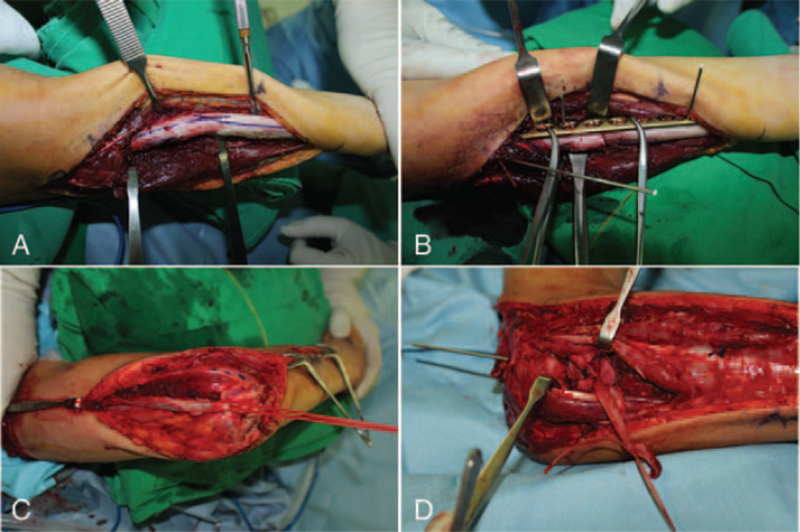
Preoperative radiographs performed at 16 mo after injury. Radiographs showing an anterior convex deformity of 20° and medial convex deformity of 21° at the radial fracture site indicated anterolateral dislocation of the radial head.

**Figure 5:**
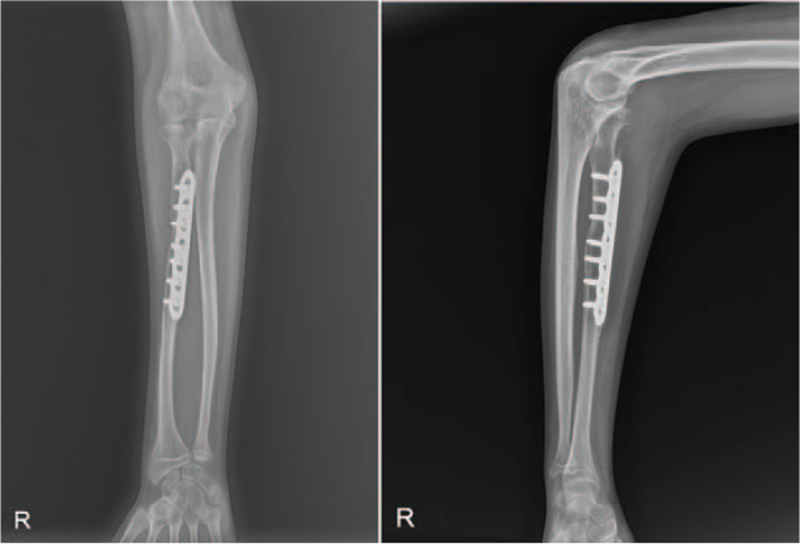
Surgical findings of dislocation of the radial head associated with malunited radial shaft. A) The proximal radius was approached between supinator and pronator teres muscles. B) After a corrective osteotomy at the malunion site, the shaft of the radius was fixed with a plate and 7 screws. C, D) A Bell Tawse procedure was performed by taking a central strip of the triceps tendon and anchoring it around the radial neck to reconstruct the annular ligament.

**Figure 6:**
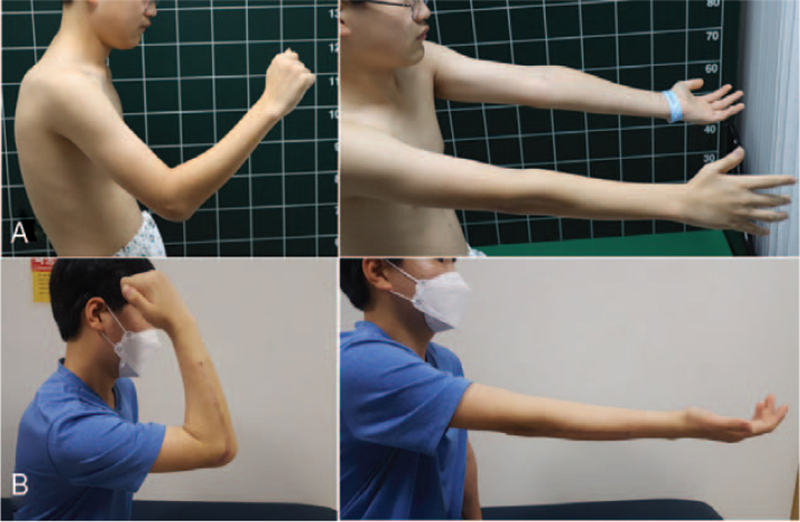
Radiographs taken at 2 yrs following the surgery. At the last follow-up, the delayed RHD associated with malunion of the radial shaft was corrected. RHD = radial head dislocation.

Sixteen months after the injury, he was referred to our hospital with complaints of restricted right forearm rotation and unreduced RHD. Radiographs showed an anterior convex deformity of 20° and a medial convex deformity of 21° at the radial fracture site, indicating anterolateral dislocation of the radial head (Fig. 4). Magnetic resonance imaging revealed mild asymmetric joint space narrowing and depression of the posterior aspect of the radial head. Manual reduction of the radial head was not possible, even with forearm supination and pronation. Corrective osteotomy was performed at the malunion site with open reduction of the radial head. The elbow joint was approached laterally. The annular ligament is extremely thin, making it difficult to recognize. The radial head is dislocated anterolaterally. The joint surface showed mild degeneration. The shape of the radial head suggests slight hypertrophy. Posterolateral articular cartilage of the radial head appeared to be worn. The proximal radius was approached between the supinator and pronator teres muscles (Fig. 5A). The posterior interosseous nerve was identified and protected. After radial osteotomy at the malunion site, a trapezoidal portion of the radial shaft of 1 cm in length was removed and replaced upside down at the osteotomy site. This corrected the angulation deformity precisely with a 5 mm longitudinal shortening of the radius. The shaft of the radius and bony autograft were fixed with a plate and 7 screws (Fig. 5B). Although forearm rotation caused subluxation of the radial head, the radial head was anatomically reduced. An incision of approximately 5 cm was made obliquely from the proximal part of the existing incision (Fig. 5C). The Bell Tawse procedure was performed by placing a central strip of the triceps tendon and anchoring it around the radial neck to reconstruct the annular ligament (Fig. 5D). Finally, the radial head was reduced using transarticular Kirschner wires with the elbow in 90° flexion and the forearm in neutral rotation. After cast immobilization for 3 weeks, active elbow flexion-extension was started with the removal of the Kirschner wires. Active forearm rotation was permitted 5 weeks after surgery. Two years after surgery, the delayed RHD associated with malunion of the radial shaft was corrected (Fig. 6). The flexion of the right elbow was 105° with an extension of 0°. Supination was 85° and pronation was 60° in the right forearm (Fig. 1B). The patient did not complain of difficulty in performing tasks such as washing the face, eating something, or cleaning the perineal area after bowel movement.

## 4 Discussion

Radial head dislocation (RHD) is generally caused by hyper-pronation injury with sequential disruption of the annular ligament, quadrate ligament, and interosseous membrane at the time of trauma.^[1]^ Regardless of the onset (acute, delayed, or chronic), most cases of RHD are caused by Monteggia fracture-dislocation associated with fracture of the ipsilateral ulna. RHD with concomitant radial shaft fractures is rare. Evans et al^[6]^ reported that fractures may occur in the proximal third of the radial shaft when additional pronation force is applied while the radial head is dislocated. Meanwhile, RHD due to malunion of an isolated fracture of the radial shaft is extremely rare in the English-language literature. To date, only 2 cases have been reported. Yasutomi et al^[4]^ reported a recurrent anterior dislocation with malunion of the radial fracture 3 years after injury. The radial shaft showed an anterior convex deformity. The radial head was reduced in supination, but dislocated in pronation. Yamazaki et al^[5]^ reported an RHD occurring 6 weeks after injury as a result of malunion of the ipsilateral radial shaft in a 12-year-old girl.

In the present case, a 12-year-old boy was diagnosed with an isolated radial shaft fracture at the time of the injury. A long arm cast was applied, and immobilization was performed for 6 weeks.

It is improbable that the current study was a case with a missed dislocated radial head because no dislocation of the radial head was seen on radiographs taken continuously until 6 weeks after injury. The patient stated that he was able to use his right arm for approximately 10 months without any major inconvenience in daily activity, although his symptoms had worsened in the last 6 months. Preoperative radiographs revealed mild osteoarthritis around the radial head and eccentric hypertrophy of the head. Late osteoarthritis of the radiocapitellar joint may be due to an eccentric load applied to the joint with a long-standing malunion of the radius. We believe that when the forearm is pronated further, the uppermost part of the malunited site of the radius might have impinged on the ulna and acted as a pivot so that further pronation of the forearm produces rotational and translational torque on the radial head. Such a torque would easily pull the radial head out distally from the damaged annular ligament.

Meanwhile, shaft fractures of the radius or ulna are one of the few pediatric fractures that show a real risk of complications and prolonged morbidity. Nearly 60% of children with a middle-third forearm fractures show residual ROM in the forearm as complications related to length discrepancy, residual malangulation, malrotation deformity, and interosseous contracture.^[7]^ Tarr et al^[8]^ have reported that angular deformity of 20° in the radius can result in 30% or greater decrease in pronation and/or supination in their cadaver studies. Therefore, the most important aim of treatment is to restore the rotational range of motion in the forearm in the long term, while minimizing complications. However, because angular deformation of the radial shaft corrects spontaneously, not more than 1° in a year until skeletal maturity, nonoperative treatment can be considered only for angulations of 5° to 10° or less for those aged 8 years or older.^[7]^ If treated non-operatively, radiographic follow-up is recommended at 1, 2, and 3 weeks postoperatively to prevent malunion. Healing with a satisfactory callus allows for light mobilization. However, heavy sports should not be allowed for several months because the risk of refracture increases during the next 4 to 6 months.^[9]^ In the current case, radiographs performed at the time of injury showed a linear fracture without displacement of the radius at the proximal third, with an anterior angulation deformity of 8°. The anterior convex deformity increased to 16° 6 weeks after the injury. We believe that it is necessary to recognize increased deformity by performing regular radiographs at the early part of the injury and attempt to reduce angulation by changing the molding of the cast. In addition, the patient stated that he had practiced sports activities during the early stages of bone union. Since remodeling ability is reduced at this age, sports activity should not be allowed for several months.

Delayed RHD secondary to malunion of radial shaft fractures is very rare. Currently, there is no widely accepted surgical treatment method. The most important aim of treatment is to restore the ROM of the elbow by correcting the deformity and reducing dislocation. Yasutomi et al^[4]^ reported that a closing wedge osteotomy was performed on the malunion at the mid 1/3 of the radial shaft 3 years after injury. However, reconstruction of the annular ligament and correction of the radial head inclination were not performed. Yamazaki et al^[5]^ performed corrective osteotomy 3 months after injury and found that the annular ligament was repaired.

In the present case, 16 months after injury, corrective osteotomy was performed at the site of malunion with an open reduction of the radial head using an extensile lateral approach. The annular ligament was disrupted. In addition, forearm rotation causes radial head subluxation. Therefore, reconstruction of the annular ligament is necessary. The Bell Tawse procedure was originally introduced to treat malunited anterior Monteggia fractures.^[10]^ He described the reconstruction of the annular ligament by turning down a strip of the triceps tendon and anchoring it around the radial neck. Lloyd-Roberts et al^[11]^ reported that triceps slip is preferable to palmaris tendon grafting because it avoids unnecessary donor site morbidity. Annular ligament reconstruction was also performed using a strip of the triceps tendon via an extensile lateral approach. At the last follow-up, delayed RHD associated with malunion of the radial shaft was corrected, and the boy showed good improvement in performing movements such as washing the face, eating something, and cleaning the perineal area after a bowel movement.

In conclusion, annular reconstruction and corrective osteotomy of the radius using an extensile lateral approach are useful for treating delayed RHD secondary to malunion of isolated radial shaft fractures.

## Author contributions

**Conceptualization:** Sung Il Wang.

**Data curation:** Seung Cheol Lee.

**Formal analysis:** Seung Cheol Lee.

**Supervision:** Sung Il Wang.

**Writing – original draft:** Sung Il Wang.

**Writing – review & editing:** Sung Il Wang.
